# Secular trends in health-related physical fitness among 11–14-year-old Croatian children and adolescents from 1999 to 2014

**DOI:** 10.1038/s41598-021-90745-y

**Published:** 2021-05-26

**Authors:** Mario Kasović, Lovro Štefan, Vilko Petrić

**Affiliations:** 1 Department of General and Applied Kinesiology, Faculty of Kinesiology, University of Zagreb, Horvaćanski zavoj 15, Zagreb, Croatia; 2 Department of Sport Motorics and Methodology in Kinanthropology, Faculty of Sports Studies, Masaryk University, Brno, Czech Republic; 3 Department of Educational Studies, Faculty of Teacher Education, University of Rijeka, Rijeka, Croatia

**Keywords:** Health care, Health occupations, Risk factors

## Abstract

The main purpose of the study was to analyze secular trends of health-related physical fitness in 7–14-year-old Croatian children and adolescents from 1999 and 2014. In this observational cross-sectional study, we recruited 5077 children and adolescents between ages 11 and 14 (50.8% girls) from five primary schools located in the capital city of Zagreb. Physical fitness performance was tested from 1999 until 2014. Physical fitness performance included: (1) body-mass index (measure of body size), (2) standing broad jump (measure of lower-body power), (3) polygon backwards (measure of general coordination and agility), (4) sit-ups in 60 s (measure of upper-body strength), (5) sit-and-reach test (measure of flexibility) and (6) 6-min run test (measure of cardiorespiratory fitness). Boys performed better in all physical fitness tests, except for sit-and-reach test (*p* < 0.001). In boys, between 1999 and 2014, body size, upper-body strength and coordination/agility increased, while flexibility, lower-body power and cardiorespiratory fitness decreased. During the same period, girls experienced an increase in body size, lower-body power, upper-body strength, coordination/agility and flexibility, while cardiorespiratory fitness decreased. This study shows that cardiorespiratory fitness, flexibility and coordination/agility decrease, while upper-body strength increases in both sexes. These findings should serve as an avenue for national monitoring system to screen and track biological development in children and adolescents.

## Introduction

Physical fitness is an important and one of the most relevant indicators of health and sports participation in young populations^1–3^. Evidence suggests that higher levels of physical fitness in childhood may prevent from developing some communicable and non-communicable diseases in adulthood^2,4,5^. Apart from general physical fitness, separate components of physical fitness (body composition, cardiorespiratory, muscular and motor) have been inversely associated with cardiovascular and metabolic diseases, stroke and all-cause mortality^6–8^.

Despite the fact that risk factors for diseases usually manifest during adulthood, studies have shown that childhood and adolescence are critical time-points for maintaining ‘good’ lifestyle habits and develop high physical fitness levels^9^ due to well-tracking characteristics of physical fitness from childhood to adulthood^1^. However, a common perception has been embraced that the physical fitness in youth has declined because of more time spent in sedentary behavior, lack of physical activity and consuming fat-rich food^10^. Such decrease is, however not uniform in different countries and physical fitness modalities^11,12^.

Secular trends of physical fitness in children and adolescents have been well-studied in the past decades^10,12–35^. In general, values in cardiorespiratory, muscular and flexibility fitness decline, and values in body-mass index (as a measure of body size) increase, especially in work that goes back further in time (between 1960 and 1980s), while there have been conflicting findings for motor fitness in terms of a decrease, increase or stagnation^36^. Such inconsistencies may depend on the sample size, age- and sex-specific populations studied, and physical fitness tests applied^12^.

In recent years, several European countries have proposed national monitoring systems for screening and monitoring biological development of children and adolescents^10,37–39^. Although such measures through projects (like the HBSC study) have been implemented and tested in Croatian youth, the most recent study has shown that ≈ 50% of youth in Croatia do not meet the recommended levels of physical activity, ≈ 25% exceed 2 h of screen time per day and have high blood pressure, and ≈ 20% have excess weight, leading to overweight and obesity status^40^. Thus, being able to keep tracking separate components of physical fitness would benefit from establishing national-wide policies and strategies aiming to standardize the assessment of children's somatic development and functional capacity in critical periods of childhood and adolescence^10^.

Therefore, the main purpose of the study was to analyze secular trends of health-related physical fitness in 7–14-year-old Croatian children and adolescents from 1999 and 2014. Based on previous evidence, we hypothesized that poorer values would be observed for body size (an increase), cardiorespiratory fitness and flexibility (a decrease), with improvements in muscular fitness.

## Methods

### Study participants

In this observational cross-sectional study, we recruited 5,077 children and adolescents between ages 11 and 14 (50.8% girls) from five primary schools located in the capital city of Zagreb. To be included, students had to be healthy and participated in physical education classes at the time of study and had to be not specifically trained for performance in the administered tests^12^. Physical fitness performance was tested from 1999 until 2014 in different sex- and age-specific samples of children and adolescents. The study was approved by the Faculty of Kinesiology, University of Zagreb, Croatia 8 ethical code number: 02/2014). Parent of each participant and all participants gave informed written consent before enrollment into the study for participation and approving to publish the information in an online open access publication. Analyses and procedures performed in the study were conducted in accordance with the Declaration of Helsinki^41^. The tests were performed each year in September.

### Health-related physical fitness

Height and weight were objectively measured in light clothing by using stadiometer and digital scale with a precision of 0.1 cm and 01 kg. *Body-mass index* was calculated by dividing weight in kg with height in m^2^ [weight(kg)/height(m)^2^]. *Standing broad jump* tests jumping distance from a standing start (‘frog leap’). While performing the jumps, each child was asked to bend their knees with their arms in front of them, parallel to the ground, then to swing both arms, push off vigorously and jump forward as far as possible, trying to land with their feet together and stay upright^42^. *Polygon backwards* tests motor coordination and agility, where the subject had to crawl backwards and cover a 10-m distance. The starting position for the children was behind the starting line as the children faced backwards on all fours. The tester gave the starting signal. The test included backward crawling (A) over and (B) under the 35 cm high obstacles that were placed at (A) 3 m and (B) 6 m from the starting line. The task was measured in tenths of a second (0.1 s)^43^. *Sit-up test* evaluates abdominal muscles function as number of sit-ups completed from lying position (knees bent at a 90°) in 60 s. Children were seated on the floor, backs straight, hands clasped behind their neck, knees bent at 90° with heels and feet flat on the mat. They then lay down on their backs, shoulders touching the mat, and returned to the sitting position with their elbows out in front to touch their knees, keeping the hands clasped behind their neck the whole time^42^. *Sit-and reach test* assesses the level of flexibility. By sitting on the floor or a mat, legs straight under the angle of 90°, the person being tested reached forward with the arms (hands overlapping). The distance of reach was measured in centimeters using a measuring non-elastic tape attached on the floor^44^. *6-min run test* is used to assess the level of cardiorespiratory fitness. Participants were asked to run or walk constantly for 6 min. The distance covered by each participant was measured by test leaders^24^.

### Data analysis

Basic descriptive statistics are presented as mean and standard deviation (SD). Kolmogorov–Smirnov test was applied to identify outliers, which were subsequently excluded. Analysis of variance (ANOVA) was used to determine the differences between sexes in all physical fitness components. To assess the associations between the assessment year and each physical fitness aspect, we used generalized estimating equations. The working correlation matrix was set to exchangeable in all analyses. The regression models were tested for several assumptions: (1) multicollinearity diagnostics using variance inflation index, (2) normality of residuals using the normal probability plot and histogram of residuals, and (3) heteroscedasticity using the standardized residuals vs. predicted plot. All assumptions were met for all regression models. Cohen’s *d* effect size calculations were performed to compare the magnitude of difference between 1999 and 2014, with thresholds set at 0.2, 0.21–0.5, 0.51–0.8, and 0.8 for trivial, small, moderate, and large magnitudes of effect, respectively. Sex-specific analyses were performed, since there were significant differences between boys and girls in all physical fitness tests (*p* < 0.05). Two-sided *p*-values were used, and significance was set at α < 0.05. All the analyses were calculated in Statistical Packages for Social Sciences v.23 (SPSS, Chicago, IL, United States).

### Ethics approval and consent to participate

The study was approved by the Faculty of Kinesiology, University of Zagreb, Croatia. The informed consent voluntarily was signed by the participants, participants’ parents or their guardians.

### Consent for publication

The informed consent voluntarily was signed by the participants, participants’ parents or their guardians.

## Results

Basic descriptive statistics are presented in Table 1. Boys had higher body-mass index value (*p* < 0.001) and performed better in standing broad jump (*p* < 0.001), polygon backwards (*p* < 0.001), sit-ups in 60 s (*p* < 0.001) and 6-min run test (*p* < 0.001), compared to girls. Girls scored better in sit-and-reach test (*p* < 0.001).

**Table 1:** Basic descriptive statistics of the study participants (*N* = 5077).

Sex/year	Health-related physical fitness components					
	Body-mass index (kg/m^2^)	Standing broad jump (cm)	Polygon backwards (sec)	Sit-ups in 60 s (#)	Sit-and-reach test (cm)	6-min run test (m)
	Mean (SD)	Mean (SD)	Mean (SD)	Mean (SD)	Mean (SD)	Mean (SD)
**Boys**						
1999	18.7 (2.7)	175.6 (26.7)	12.2 (3.9)	35.1 (7.9)	51.0 (10.6)	1173.7 (194.5)
2000	18.6 (2.8)	172.3 (24.8)	13.8 (4.5)	38.7 (8.3)	57.2 (14.1)	1168.5 (174.0)
2001	18.8 (2.4)	172.1 (25.4)	13.5 (4.2)	38.7 (7.4)	55.2 (13.8)	1142.4 (168.3)
2002	18.9 (2.6)	171.2 (20.8)	13.3 (3.8)	38.9 (6.9)	54.4 (9.9)	1109.3 (123.9)
2003	19.1 (2.9)	175.8 (21.5)	12.5 (3.6)	39.4 (7.9)	62.0 (10.0)	1175.4 (172.3)
2004	19.5 (3.4)	171.8 (23.5)	13.0 (4.0)	41.2 (8.8)	64.4 (11.8)	1179.1 (170.2)
2005	19.9 (4.0)	175.5 (25.6)	14.6 (4.4)	38.2 (8.4)	63.5 (11.8)	1144.6 (191.3)
2006	20.2 (4.0)	171.8 (27.3)	15.0 (4.6)	37.4 (8.5)	54.8 (9.7)	1168.5 (194.2)
2007	20.1 (4.2)	169.3 (27.3)	15.5 (5.1)	36.8 (7.5)	56.6 (11.1)	1144.4 (199.9)
2008	20.0 (3.8)	165.6 (24.7)	15.4 (4.5)	37.0 (7.3)	55.4 (11.5)	1138.7 (178.2)
2009	19.8 (3.7)	170.1 (25.1)	14.5 (4.3)	38.7 (8.1)	61.1 (14.3)	1085.3 (220.1)
2010	19.8 (3.2)	170.1 (29.1)	14.2 (3.5)	39.2 (8.4)	53.6 (9.2)	1164.9 (187.9)
2011	20.1 (3.3)	172.7 (27.1)	15.5 (4.8)	40.1 (8.6)	58.7 (11.5)	1131.5 (221.8)
2012	19.9 (3.3)	175.7 (26.3)	13.0 (3.1)	41.2 (8.2)	57.2 (11.4)	909.3 (120.5)
2013	20.1 (3.2)	174.6 (29.8)	11.1 (1.1)	41.2 (8.5)	44.3 (9.6)	846.9 (110.8)
2014	20.1 (3.5)	169.9 (21.9)	10.9 (1.1)	40.7 (8.1)	44.7 (9.2)	828.3 (140.7)
Total	19.6 (3.5)	172.3 (25.6)	13.7 (4.2)	38.9 (8.3)	56.4 (12.6)	1029.2 (175.9)
**Girls**						
1999	19.0 (3.1)	156.0 (23.8)	17.0 (5.8)	33.3 (8.9)	57.0 (11.4)	1108.5 (220.3)
2000	18.3 (2.5)	163.1 (21.1)	14.5 (3.5)	33.4 (7.5)	66.5 (14.9)	1014.8 (159.1)
2001	19.3 (3.3)	148.3 (24.2)	22.4 (9.4)	31.3 (10.2)	55.7 (11.9)	1087.4 (276.0)
2002	18.6 (2.5)	159.4 (18.9)	16.4 (4.7)	33.4 (5.1)	61.2 (9.9)	986.9 (103.2)
2003	19.3 (3.0)	160.7 (21.6)	14.9 (4.5)	34.0 (7.2)	71.5 (11.2)	1017.3 (149.7)
2004	19.5 (4.0)	160.9 (23.1)	14.8 (5.5)	36.1 (8.7)	72.6 (11.9)	1032.0 (144.4)
2005	19.2 (3.3)	161.2 (20.7)	15.7 (5.7)	36.8 (7.9)	72.6 (11.7)	1070.7 (134.7)
2006	19.0 (3.2)	159.8 (22.1)	16.4 (5.5)	35.8 (7.6)	63.9 (11.0)	1067.6 (140.1)
2007	19.0 (2.9)	160.0 (23.5)	17.2 (4.5)	34.9 (7.4)	63.3 (13.5)	1040.3 (133.3)
2008	18.8 (2.8)	152.0 (22.3)	18.2 (5.1)	33.0 (7.4)	59.5 (11.0)	1022.0 (133.9)
2009	19.1 (2.9)	154.4 (21.4)	16.5 (4.5)	35.2 (5.9)	65.7 (12.5)	942.0 (164.5)
2010	18.7 (2.4)	160.8 (21.8)	15.6 (4.6)	36.1 (6.8)	67.8 (12.6)	1017.3 (186.8)
2011	18.9 (2.6)	163.9 (22.0)	16.2 (4.2)	37.5 (6.3)	67.0 (11.8)	1038.2 (160.5)
2012	18.9 (3.1)	161.5 (21.4)	14.3 (3.8)	37.2 (6.3)	64.6 (11.7)	848.3 (148.5)
2013	19.0 (3.0)	160.6 (23.5)	11.6 (0.9)	38.2 (6.4)	51.7 (9.4)	797.9 (130.5)
2014	19.1 (2.9)	161.2 (20.4)	11.6 (1.0)	39.3 (7.0)	52.0 (9.8)	680.5 (128.7)
Total	19.0 (3.0)	158.4 (22.6)	16.3 (5.9)	34.9 (8.0)	63.2 (13.6)	973.1 (156.1)

**Figure 1:**
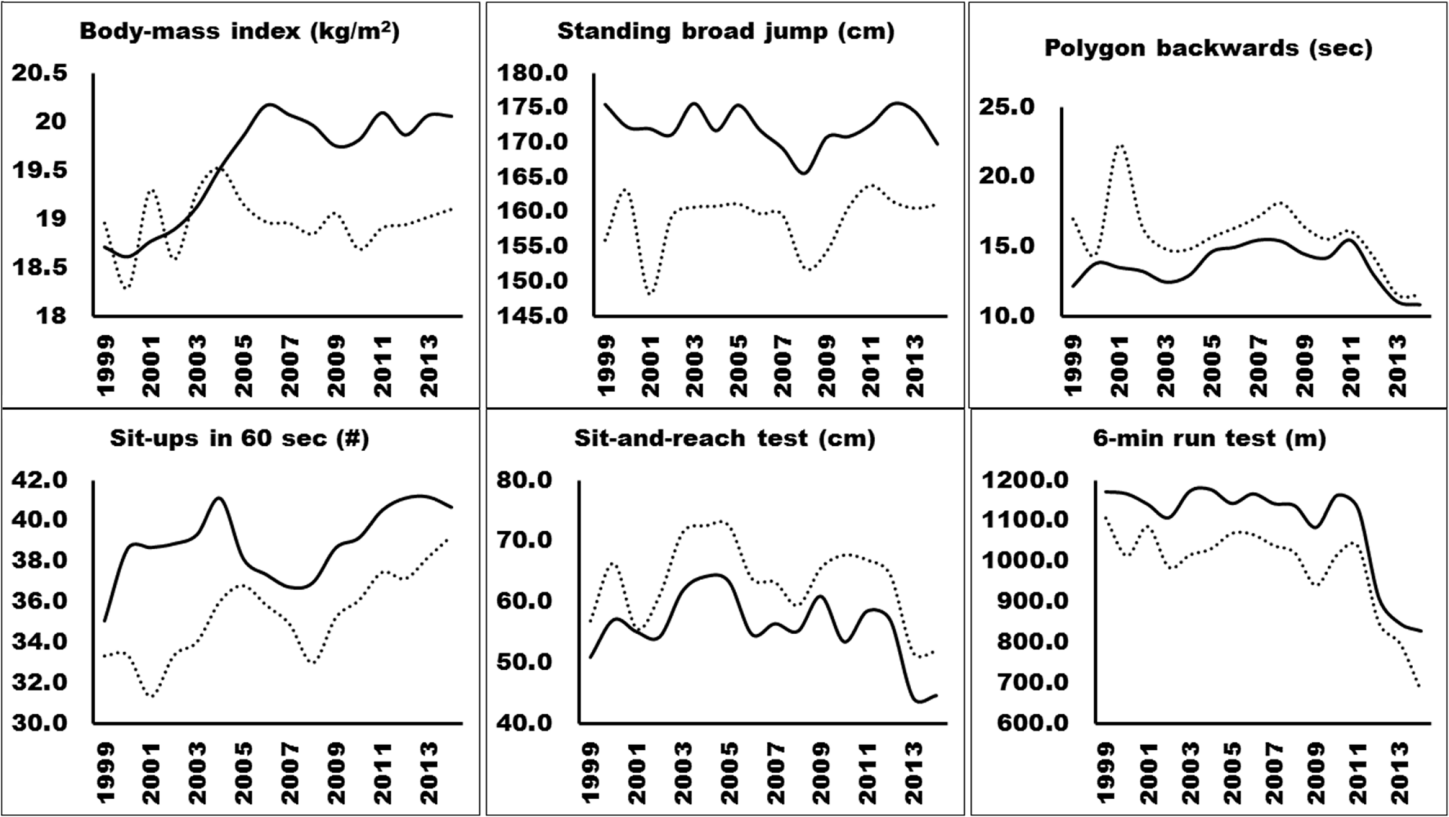
Secular trends of in health-related physical fitness among 11 to 14-year-old boys (black full line) and girls (discontinuous line) from 1999 to 2014.

Secular trends in health-related physical fitness are presented in Fig. 1. From 1999 to 2014, body-mass index and the number of sit-ups in 60 s increased by 7.5% (standardized *β* = 0.12, *p* < 0.001, ES = 0.45) and 15.9% (standardized *β* = 0.13, *p* < 0.001, ES = 0.70), while the results in standing broad jump, polygon backwards, sit-and-reach test and 6-min run test decreased by 3.2% (standardized *β* = − 0.02, *p* = 0.327, ES = 0.23), 10.7% (standardized *β* = − 0.01, *p* = 0.939, ES = 0.15), 12.4% (standardized *β* = − 0.14, *p* < 0.001, ES = 0.61) and 12.3% (standardized *β* = − 0.55, *p* < 0.001, ES = 2.00) in boys, respectively. During the same period, girls experienced a significant increase in standing broad jump and sit-ups in 60 s by 3.3% (standardized *β* = 0.07, *p* = 0.004, ES = 0.23) and 18.0% (standardized *β* = 0.21, *p* < 0.001, ES = 0.75), while the results in polygon backwards, sit-and-reach test and 6-min run test decreased by 31.8% (standardized *β* = − 0.20, *p* < 0.001, ES = 1.30), 8.8% (standardized *β* = 0.03, *p* = 0.268, ES = 0.47) and 38.6% (standardized *β* = − 0.52, *p* < 0.001, ES = 2.37), respectively.

## Discussion

The main purpose of the study was to analyze secular trends of health-related physical fitness in 7–14-year-old Croatian children and adolescents from 1999 and 2014. The main findings are: (1) boys tend to perform better in upper-body strength tests, yet decrease the results in flexibility and cardiorespiratory fitness tests, (2) girls tend to perform better in upper-body strength, lower-body power and coordination/agility tests, yet decrease the results in cardiorespiratory fitness test.

Previous evidence has shown that cardiorespiratory performance declines in children and adolescents^12,16–19^. Our results confirm a negative trend of aerobic fitness in both sexes, which is alarming because low cardiorespiratory fitness is associated with development of numerous diseases and all-cause mortality^1–9^. It has been well-documented, that cardiorespiratory fitness correlates with physical activity^45^. In Croatia, studies have shown a negative trend of meeting the recommended levels of physical activity from 2002 to 2010^46^, possibly causing a significant decline in cardiorespiratory fitness. Such findings have been obtained previously^12^, pointing out that by enhancing cardiorespiratory fitness, one would be expecting to increase the level of physical activity and vice versa. Of note, we observed that the level of cardiorespiratory fitness had declined from 1999 to 2014 by ≈ 22.0% for both sexes, which is similar to previous studies using 20-m shuttle run test as a proxy of cardiorespiratory fitness^16^. In general, a recent systematic review aiming to report trends in physical fitness has shown, that the declining trend in cardiorespiratory fitness is more obvious in studies with more time periods or longer follow-ups^36^, which has also been supported by large-scale epidemiological findings^18^.

The observation of a declining trend of flexibility in this study is consistent to previous studies conducted among similar age groups^12,21,23,32,36,47^. Flexibility performance is associated to body size (mainly to body-mass index), and since we observed a significant increase of body-mass index from 1999 to 2014, it is possible that such increase would correlate to a negative trend of flexibility^21^. Indeed, there has been a general consensus that flexibility in children and adolescents steadily declines, which has been proven previously^36^. Being able to support such statement comes from similar methodological testing procedure and using sit-and-reach test, as a proxy of flexibility.

In both sexes, we observed a positive trend of upper-body strength (by using the number of sit-ups in 30 s), which is consistent to previous findings^11,12,23,48,49^. On the other hand, lower-body power had been stagnating between 1999 and 2014, while in girls a significant positive trend was observed^48,49^. Specifically, studies by Albon et al.^48^ and Huotari et al.^49^ showed no significant changes for standing broad jump performance. On the other hand, a large-scale systematic review conducted among 27 countries and 6–19-year-old children and adolescents has shown that power ability had been slightly improving between 1958 and 2003, regardless of gender, age or socioeconomic status^11^. The discrepancy between this and previous studies may be explained by using different sample sizes, age-groups, statistical analyses and time-periods for assessing secular trends. According to recent systematic review^36^, the majority of the primary studies reported a decline in strength performance. Although we found a significant positive change of standing broad jump performance in girls, the effect size between 1999 and 2014 was only trivial.

Coordination/agility performance assessed through polygon backwards had been improving in both sexes, with significantly greater changes in girls. Such findings are similar to previous studies^12,24^. However, a study by Eberhardt et al.^36^ showed that the same number of studies reported a decrease, increase or stagnation. The inconsistency between the studies comes from the construct of coordinative abilities, which are being complex and multidimensional^36^. Second, different coordination tasks can measure different aspects of coordination^36^. Therefore, it is difficult to compare the findings in this measure, since different motor tests to assess coordination/agility (obstacle course, balancing backwards, target throwing or one-leg stand) have been used previously^36^. Nevertheless, polygon backwards is a reliable and valid test to assess the level of coordination/agility in children^42^.

Finally, an increased trend in body-mass index of this study is consistent with findings from previous studies^12,13,21,50^. The ecological shift towards not meeting the recommended levels of physical activity, exceeding 2 h of screen time per day and having high blood pressure and excessive weight in Croatian youth^40^ may have led to a negative trend of body-mass index, particularly in boys. However, stronger trend in boys observed in this study may be attributed to different body composition levels between sexes, where boys have higher values of lean-muscle mass and lower values of fat-mass, compared to girls. Unfortunately, body-mass index cannot discriminate between lean-muscle mass and fat mass and, therefore, changes in body size need to be interpreted with caution.

This study has several limitations. First, by using a cross-sectional design, we cannot determine the causality of the association. Second, we used the 6-min run test as a proxy of cardiorespiratory fitness. By using more objective methods, like the treadmill test, the findings might have been different. Also, the time needed for polygon backwards was measured manually and not with the use of a light barrier. Finally, each test in a specific year was performed only once, limiting maximal efficiency and performance. Therefore, future studies should screen and monitor the level of physical fitness during the follow-up period by using standardized tests performed a few times, in order to determine separate and overall physical fitness trends.

The study confirms the hypothesis of previous evidence, that cardiorespiratory fitness has negative secular trend, yet upper-body strength tends to have positive secular trend in both sexes. Boys and girls seem to produce different secular trends in other physical fitness components, pointing out that sex-specific surveillance system of physical fitness should be incorporated within the school system, in order to monitor and track separate and overall physical fitness in children and adolescents.

## Data Availability

The datasets used and/or analyzed during the current study are available from the corresponding author on reasonable request.
